# High Malnutrition Rate in Venezuelan Yanomami Compared to Warao Amerindians and Creoles: Significant Associations WITH Intestinal Parasites and Anemia

**DOI:** 10.1371/journal.pone.0077581

**Published:** 2013-10-15

**Authors:** Lilly M. Verhagen, Renzo N. Incani, Carolina R. Franco, Alejandra Ugarte, Yeneska Cadenas, Carmen I. Sierra Ruiz, Peter W. M. Hermans, Denise Hoek, Maiza Campos Ponce, Jacobus H. de Waard, Elena Pinelli

**Affiliations:** 1 Laboratorio de Tuberculosis, Instituto de Biomedicina, Universidad Central de Venezuela, Caracas, Venezuela; 2 Laboratory of Pediatric Infectious Diseases, Radboud University Medical Center, Nijmegen, The Netherlands; 3 Departamento de Parasitología, Facultad de Ciencias de la Salud, Universidad de Carabobo, Valencia, Venezuela; 4 Departamento de Pediatría, Hospital de Niños ‘J.M. de los Ríos’, Caracas, Venezuela; 5 Escuela de Bioanálisis, Universidad Central de Venezuela, Caracas, Venezuela; 6 Center for Infectious Disease Control Netherlands, National Institute for Public Health and the Environment (RIVM), Bilthoven, The Netherlands; 7 Department of Health Sciences, VU University, Amsterdam, The Netherlands; Instituto de Higiene e Medicina Tropical, Portugal

## Abstract

**Background:**

Children in rural areas experience the interrelated problems of poor growth, anemia and parasitic infections. We investigated the prevalence of and associations between intestinal helminth and protozoan infections, malnutrition and anemia in school-age Venezuelan children.

**Methods:**

This cross-sectional study was conducted in 390 children aged 4-16 years from three rural areas of Venezuela: the Amazon Region, Orinoco Delta and Carabobo State. Stool samples were collected for direct parasitic examinations. Anthropometric indicators of chronic (height-for-age Z score) and acute (weight-for-height and Body Mass Index (BMI)-for-age Z score in respectively children under 5 years of age and children aged 5 years and above) malnutrition were calculated. Multivariate linear and logistic regression models were built to determine factors associated with nutritional status and polyparasitism.

**Results:**

Hookworm and *Strongyloides stercoralis* prevalences were highest in children from the Amazon rainforest (respectively 72% and 18%) while children from the Orinoco Delta and Carabobo State showed higher rates of *Ascaris lumbricoides* (respectively 28% and 37%) and *Trichuris trichiura* (40% in both regions). The prevalence of *Giardia lamblia* infection was not significantly different between regions (average: 18%). Anemia prevalence was highest in the Amazon Region (24%). Hemoglobin levels were significantly decreased in children with a hookworm infection. Malnutrition was present in respectively 84%, 30% and 13% of children from the Amazon Region, Orinoco Delta and Carabobo State. In multivariate analysis including all regions, *G. lamblia* and helminth infections were significantly and negatively associated with respectively height-for-age and weight-for-height/BMI-for-age Z scores. Furthermore, hemoglobin levels were positively associated with the height-for-age Z score (0.11, 95% CI 0.02 - 0.20).

**Conclusions:**

In rural populations in Venezuela helminthiasis and giardiasis were associated with acute and chronic nutritional status respectively. These data highlight the need for an integrated approach to control transmission of parasites and improve the health status of rural Venezuelan children.

## Introduction

One-third of the world’s population harbors at least one species of intestinal parasite with school-age children bearing the greatest burden in terms of morbidity and mortality [1,2]. Parasitic infections are an important cause of nutritional and energetic stress. The most significant cause of nutritional stress resulting from parasitic infections is hookworm-associated iron deficiency anemia. In a recently performed survey in Kenya including 16,941 children aged 5 to 16 years, the prevalence of anemia was as high as 35% and low hemoglobin levels were significantly associated with hookworm infections [3]. Eosinophilia can also occur secondary to parasitic infections. Infection with intestinal helminths results in immune responses involving cytokines produced by T helper 2 (Th2) cells and consequently the development of eosinophilia [4]. Even in the absence of overt disease, long-lived helminth and protozoan infections may cause immune alterations and nutritional stress associated with immunosuppression, poor growth patterns and chronic malnutrition, i.e. stunting [5-7]. For intestinal helminths, the morbidity severity depends on the intensity of infection [8-10]. Stunting is associated with impaired physical growth and cognitive development [11]. Intestinal parasitic infections have also been associated with acute malnutrition, also known as wasting [12-14]. Wasting results from inadequate nutrition over a shorter period and carries an immediate increased risk of morbidity and mortality [15]. Stunting and wasting can have different determinants and respond to different interventions. Therefore, consideration of stunting and wasting as anthropometric indicators is more useful than consideration of underweight, which encompasses both stunting and wasting [16]. 

Parasitic infections typically afflict the poorest population segments and prevalence rates of both parasitic infections as well as malnutrition are generally higher in rural compared to urban settings [14,17,18]. There has been an increased awareness of the public health importance of parasitic infections, but much of the interest has been focused on chemotherapy as a means of control while the study of social, cultural and economic factors underlying infection risk has been relatively neglected [19]. Recently, Gazzinelli and co-authors outlined a research agenda for the socioeconomic and health systems research required for the development of sustainable helminth control programs. They concluded that research on social and environmental determinants can contribute significantly to the prevention and control of helminth disease [20]. We present the results of a cross-sectional survey estimating the prevalence of intestinal parasitic infections in school-age children residing in three rural regions of Venezuela, a country with a rich cultural display and a wide variety of climate regions. Two of the three regions are inhabited by Amerindian populations whereas the third region is inhabited by a rural non-indigenous (Creole) population. In addition to providing an overview of helminth and *Giardia lamblia* infection prevalence rates in all three regions, we also studied the association of intestinal parasitic infections and anemia with acute and chronic malnutrition. To our knowledge, this is the first study comparing prevalence rates of intestinal parasites and malnutrition between three ethnically different populations inhabiting geographically dispersed areas in a South American country.

## Methods

### Ethics statement

In the Creole population in Carabobo State the nature and objectives of the study were explained to the parents of all children in Spanish. In the two Amerindian populations in the Amazon Region and the Orinoco Delta, the nature and objectives of the survey were translated into the native language of inhabitants by bilingual native interpreters. In the Amazon Region, the study was approved by the ethical committee of the Amazon Research Center for Tropical Diseases (Centro Amazónico para Investigación y Control de Enfermedades Tropicales, CAICET). The study in the Carabobo State was part of a collaboration between the Universidad de Carabobo in Valencia, Venezuela, and in The Netherlands the National Institute for Public Health and the Environment (RIVM) in Bilthoven and the VU University in Amsterdam. It was approved by both the ethical committee of the Universidad de Carabobo and the ethical board of the VU University of Amsterdam. In the Orinoco Delta, the study was approved by the ethical committee of the Instituto de Biomedicina, the Regional Health Services, and the Orinoco Delta Indigenous Health Office (Servicio de Atención y Orientación al Indígena). 

Children were enrolled if their parents or primary caregivers provided written informed consent. In Carabobo State, all parents or caregivers were literate. Illiterate parents or caregivers in the Amazon Region and the Orinoco Delta signed by means of a thumb print, for which a specific section on the informed consent form was created and approved by the respective ethical committees. Anti-helminthic and anti-protozoan treatment was provided based on the fecal examinations in all three regions.

### Socio-environmental characteristics of study regions

**Figure 1:**
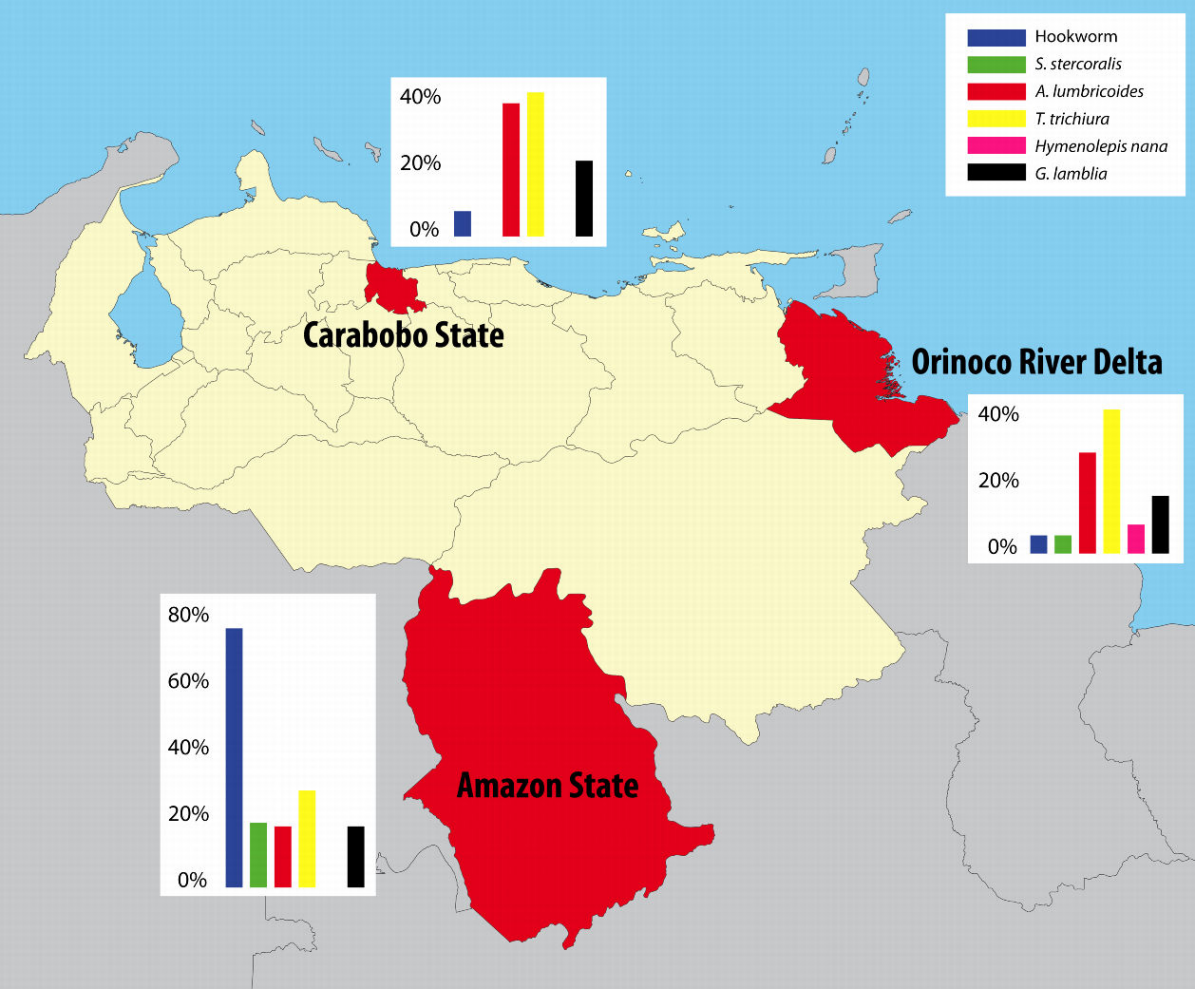
Location of study regions in Venezuela and prevalence of intestinal parasites per region.

This cross-sectional study was conducted in children aged ≥4 and <17 years from three regions of Venezuela: the Amazon Region, Carabobo State (the village of El 25) and the Orinoco Delta (Figure 1). In the Amazon Region and in Carabobo State, children were included during the dry season, respectively in November 2011 and in March 2010. Door-to-door visits were made to include age-eligible children present in the communities. If an individual was not home during the door-to-door visits, return visits were made within two weeks to include children 4-16 years of age living in the communities at the time of survey for whom parental written informed consent was obtained. In the Orinoco Delta, children were included over a period of 12 months, from May 2010 to May 2011.

The Yanomami Amerindians residing in the Amazon rainforest live in large communal houses where the entire village lives under a common roof of palm leaves called the *shabono*. Within the *shabono*, family units are incompletely separated by wooden poles. Households lack toilets and communities have no sewage facilities. The sources of drinking water in Yanomami villages are unprotected wells and river streams. Basic primary health centers are located in some of the communities, but most villages depend on visits of medical teams and do not have a constant access to healthcare. Yanomami children included in this study resided in 11 geographically dispersed villages in the High Orinoco Region of southern Venezuela, an area of perennial tropical forest intersected by a dense river system. 

El 25 is a rural community in the mountains south of Lake Valencia in Carabobo State in north-central Venezuela, inhabited by a non-indigenous (Creole) population. People in El 25 live in brick houses with toilets. However, toilet facilities are not used permanently because indoor water supply is not regular, causing a lot of people to defecate in the bushes close to their homes. Sources of drinking water are an untreated underground well and, sometimes, surface water. A small rural hospital provides villagers with basic health care facilities. 

The Orinoco Delta in northeastern Venezuela is resided by the Warao Amerindians living in wooden houses raised on stilts in small communities along the Orinoco River banks. There is no sewerage system in the Warao villages. The river is used for bathing and also for drinking and cooking. Medical help is not readily available in Warao communities; in most villages, only a small poorly equipped health post is present. Canoe trips to and from the nearest hospital can take more than 36 hours. 

### Sample collection and analysis

Fecal samples were analyzed by wet mount examinations. In each study region, duplicate slides of each sample were prepared and examined by two analysts (both experienced laboratory technicians) following standardized protocols [21]. In case of discordant results, the slides were re-read and results discussed until agreement between the technicians was reached. For quality control, triplicates of 30 randomly selected stool samples were blindly re-read by the study microscopists of all three study sites in order to check for inter-observer variability between technicians from the three regions. 

In the Amazon Region, a wet mount microscopic slide was analyzed immediately after collection of a single stool specimen to identify *G. lamblia*. Subsequently, a portion of the sample was examined using the Kato-Katz [22] and Baermann [23] methods to determine the presence of helminth eggs and larvae respectively. Intensity of infection with helminths excreting eggs was assessed by egg counts in stool and classified as light, moderate and heavy according to World Health Organization (WHO) criteria [24]. No standard larval count categories are available for *Strongyloides stercoralis* intensity, therefore arbitrary categories were defined to obtain approximately equal numbers of participants in each category: light ≤10 larvae per gram (lpg) of stool, moderate 10<lpg<30, and heavy lpg≥30.

In the Carabobo State and in the Orinoco Delta, stool samples taken on two consecutive days were preserved in sodium acetate-acetic acid formalin (SAF) [25] and stored at 4°C until examination. An aliquot of the unpreserved sample was mixed with ethanol. DNA was extracted from ethanol-preserved samples with the High Pure polymerase chain reaction (PCR) template preparation kit (Roche, Germany). Real-time PCR of fecal samples was conducted on the Roche LightCycler^®^ 480 system for determination of infection intensity categories for *Ascaris lumbricoides*, *S.* stercoralis or hookworm microscopy-positive samples [26]. Intensity categories were defined as following: light (cycle threshold (Ct) ≥35.0), moderate (30.0≤Ct<35.0) or heavy (Ct<30.0) [27].

An individual was considered to have a polyparasitic infection if more than one parasite species was identified in the fecal sample.

In the Orinoco Delta and in the Amazon Region, blood was collected from each participant into an EDTA tube for the determination of hemoglobin levels. Additionally, a peripheral blood smear was stored for microscopic leukocyte differentiation. Eosinophilia was defined as more than 5% of the total differential leukocyte count. Anemia was defined by hemoglobin levels <11.5 g/dL for children <8 years of age, <11.9 g/dL for children 8-11 years of age, <12.5 g/dL or <11.8 g/dL for respectively males and females 12-14 years of age and <13.3 g/dL or <12.0 g/dL for respectively males and females >14 years of age, in accordance with the criteria of the Centers for Disease Control and Prevention [28].

### Anthropometric measurements

Date of birth was taken from a written record when available, usually a birth certificate or a vaccination card. If birth dates were not recorded or known with certainty, the caregiver was asked to give an approximate date of birth based on a local event calendar. Height (cm) and weight (kg) were measured using WHO standards for measurement [29]. Children were measured for height and weight without shoes and in light indoor clothing by a single measurement performed by two investigators. The investigators included physicians and medical students. All were briefly trained in performing the anthropometric measurements. Height was measured using a tape measure with a precision to 0.1 cm which was fixed to a wall. Weight was measured using a calibrated electronic digital scale, with a maximum capacity of 150 kg and a precision to 0.1 kg. Scales were placed on a flat, hard surface, mostly in schools or health posts close to the children's homes. Anthropometric measurements were transformed into weight-for-height, height-for-age, and BMI-for-age Z scores based on WHO standard reference populations [30,31] using WHO anthro software [32]. Children under 5 years of age with weight-for-height or height-for-age Z scores <-2 standard deviations (SD) were defined as malnourished. Children aged ≥5 years with BMI-for-age or height-for-age Z scores <-2 SD were defined as malnourished. Weight-for-height and BMI-for-age Z scores are indicators of wasting (acute malnutrition) in respectively children under 5 years of age and those aged 5 years and above. The height-for-age Z score is an indicator for grading stunting (chronic malnutrition) in children of all ages [16,33,34].

### Statistical analysis

Categorical variables were analyzed using Chi-square test or Fisher’s exact test, as appropriate. For continuous variables, the one-way analysis of variance (ANOVA) and unpaired Student’s t tests or the nonparametric Kruskal-Wallis and Mann-Whitney’s tests were used depending on whether or not the variables were normally distributed (Kolmogorov-Smirnov’s test, p>0.05). When the variances across groups were not equal (Levene’s test p<0.05), Welch correction for non homogeneity of variance was applied. 

To determine which characteristics were associated with the nutritional status of children, multivariate linear regression models were built. To determine the factors associated with polyparasitism, a multivariate logistic regression analysis was performed. Since hemoglobin levels were only measured in children from the Amazon Region and the Orinoco Delta, for the calculations of associations with hemoglobin levels, children from the Carabobo State were excluded. Only variables with a p-value ≤0.20 in univariate analyses were considered as candidates for the multivariate models [35]. Age and sex were retained in the final multivariate models, irrespective of their p-values in univariate analysis.

## Results

Stool samples from 390 Venezuelan children aged 4-16 years residing in three regions of Venezuela were collected. A total of 264 children (68%) were infected with at least one type of parasite. Helminth infections were present in more than half of the included children in each region (Figure 1, Table 1). The prevalence rate of helminths infecting the host through skin penetration, hookworm and *S. stercoralis*, was higher in Yanomami children from the Amazon rainforest compared with the other two regions (all p<0.01). In contrast, prevalence rates of helminths that are transmitted through the fecal-oral route, *A. lumbricoides* and *Trichuris trichiura*, were lower in the Yanomami compared to the Warao children (p=0.018 and p=0.020, respectively) and the children from El 25 (p<0.01 and p=0.035, respectively). *Hymenolepis nana* was only present in the Orinoco Delta (8%). The prevalence of *G. lamblia* infections was 17%, 21% and 16% in the Amazon Region, the Carabobo State and the Orinoco Delta respectively and was not significantly different between the three regions (p=0.56, Table 1).

**Table 1:** Characteristics of included children by study region.

	**Amazon Region (n=133, 34%)**	**Orinoco Delta (n=152, 39%)**	**Carabobo State (n=105, 27%)**	**p-value**
**Demographic figures**				
Sex, n (%)				0.011
Female	50 (38)^a^ *	59 (39)^a^	58 (55)^b^	
Male	83 (62)^a^	93 (61)^a^	47 (45)^b^	
Age (years), mean (SD)	9.8 (3.1)	10.0 (3.4)	9.8 (3.9)	0.87
**Nutritional status**				
Weight-for-height Z score in children < 5 years, mean (SD) BMI-for-age Z score in children ≥ 5 years, mean (SD)	-0.26 (0.99)^a^	0.14 (1.09)^b^	0.16 (1.06)^b^	<0.01
Height-for-age Z score in children of all ages	-2.71 (1.93)^a^	-1.48 (1.20)^b^	-1.08 (0.98)^c^	<0.01
Malnourished, n (%)	101 (84)^a^	45 (30)^b^	14 (13)^c^	<0.01
**Parasitic infections, n (%)**	109 (82)^a^	93 (61)^b^	62 (59)^b^	<0.01
Helminth infections	106 (80)^a^	78 (51)^b^	55 (52)^b^	<0.01
Hookworm	96 (72)^a^	8 (5)^b^	7 (7)^b^	<0.01
*Ascaris lumbricoides*	22 (17)^a^	43 (28)^b^	39 (37)^b^	<0.01
*Strongyloides stercoralis*	24 (18)^a^	7 (5)^b^	0 (0)^c^	<0.01
*Trichuris trichiura*	36 (27)^a^	61 (40)^b^	42 (40)^b^	0.039
*Hymenolepis nana*	0 (0)^a^	12 (8)^b^	0 (0)^a^	<0.01
*Giardia lamblia*	23 (17)	24 (16)	22 (21)	0.56
**Hematologic values**				
Neutrophils (% of total WBC count), median (IQR)	49 (40 - 56)	55 (50 - 63)	not determined	<0.01
Lymphocytes (% of total WBC count), median (IQR)	33 (26 - 40)	33 (28 - 39)	not determined	0.92
Monocytes (% of total WBC count), median (IQR)	4 (2 - 8)	0 (0 - 0)	not determined	<0.01
Eosinophils (% of total WBC count), median (IQR)	11 (9 - 17)	7 (4 - 15)	not determined	<0.01
Basophils (% of total WBC count), median (IQR)	0 (0 - 0)	0 (0-0)	not determined	0.57
Hemoglobin (g/dL), mean (SD)	12.7 (1.7)	14.1 (2.4)	not determined	<0.01
Anemia, n (%)	27 (24)	14 (9)	not determined	<0.01

* Pairwise post-hoc comparisons: the same letters in the same line indicate absence of statistically significant difference.

The inter-observer variability of microscopy reading for parasitic infections was assessed on a subset of 30 fecal samples that were blindly re-read by study microscopists from the three study regions. The parasites present in the 30 randomly selected samples were hookworm, *A. lumbricoides*, *T. trichiura* and *G. lamblia*. The median kappa (κ) values for pairwise comparisons between observers showed excellent agreement for all parasites present in the samples with κ ranging from 0.81 for *T. trichiura* to 0.93 for *A. lumbricoides* (all p<0.001). 

Anthropometric measurements were obtained from 375 (96%) of the included children. The percentage of malnourished children was highest in the Amazon Region (84%) compared with both the Orinoco Delta (30%, p<0.01) and the Carabobo State (13%, p<0.01, Table 1). Multivariate linear regression analysis showed that the mean height-for-age Z score was lowest in the Amazon Region compared to the other two regions (Table 2). Males had significantly higher height-for-age Z scores than females (0.30, 95% CI 0.01-0.59). Furthermore, the height-for-age Z score was significantly negatively associated with age in years (-0.06, 95% CI -0.10 - -0.01) and *G. lamblia* infection (-0.39, 95% CI -0.76 - -0.01). Hemoglobin level was significantly positively associated with mean height-for-age Z score in multivariate analysis (0.11, 95% CI 0.02 - 0.20). Helminth infection was not significantly related to height-for-age Z score (Table 2). The indicators of acute malnutrition, weight-for-height and BMI-for-age Z score in respectively children under 5 years of age and those aged 5 years and above, also showed the lowest values in children from the Amazon Region (Table 2). Males showed a significantly higher weight-for-height or BMI-for-age Z score than females (0.22, 95% CI 0.01 - 0.44). Furthermore, the weight-for-height or BMI-for-age Z score was significantly negatively associated with the presence of a helminth infection (-0.24, 95% CI -0.46 - -0.01). *G. lamblia* infection, hemoglobin levels and age were not significantly associated with the weight-for-height/BMI-for-age Z score (Table 2).

**Table 2:** Univariate and multivariate linear regression analyses of factors associated with nutritional status.

**Characteristics**	**Height-for-age Z score in children of all ages (n=375)**		**Weight-for-height Z score in children <5 years (n=37) BMI-for-age Z score in children ≥5 years (n=338)**	
	univariate analysis	multivariate analysis	univariate analysis	multivariate analysis
	p-value	coefficient (95% CI)	p-value	coefficient (95% CI)
**Sex**	0.37		0.13	
Female		0		0
Male		0.30 (0.01 - 0.59)		0.22 (0.01 - 0.44)
**Age (years)**	0.031	-0.06 (-0.10 - -0.01)	0.085	-0.02 (-0.05 - 0.01)
**Region**	<0.01		<0.01	
Amazon Region		0		0
Orinoco Delta		1.25 (0.91 - 1.58)		0.33 (0.07 - 0.59)
Carabobo State		1.71 (1.34 - 2.08)		0.39 (0.11 - 0.67)
***Giardia lamblia* infection**	0.15	-0.39 (-0.76 - -0.01)	0.38	
**Helminth infection**	0.47		<0.01	-0.24 (-0.46 - -0.01)
**Hemoglobin (g/dL)**	<0.01	0.11 (0.02 - 0.20)	0.43	

Blood samples were collected from 111 of the 133 included Yanomami children in the Amazon Region (83%) and from 150 of the 152 included Warao children in the Orinoco Delta (99%). Almost a quarter (24%) of the Yanomami and 9% of the Warao children were anemic (p<0.01, Table 1). Hookworm infection was significantly more present in anemic children compared to non-anemic children (51% vs. 35%, p=0.043). The prevalence rates of other helminth or *G. lamblia* infections were not significantly different between children with and without anemia. Eosinophilia was significantly more often observed in children with a helminth infection compared to uninfected children (72% vs. 28%, p=0.011). In particular, children with *S. stercoralis* or hookworm infection showed significantly higher rates of eosinophilia than children not infected with these helminths (90% vs. 70%, p=0.022 and 85% vs. 65%, p<0.01, respectively). 

Since acute nutritional status and anemia were associated with intestinal helminths, we investigated their relationship with the intensity of helminth infection (Table 3). Most children (65%) infected with hookworm, *A. lumbricoides* or *S. stercoralis* had light-intensity infections. The mean hemoglobin level in children infected with hookworm was 12.8 (95% CI 12.5 - 13.2) compared to 14.0 (95% CI 13.6 - 14.3) in children not infected with hookworm. There were no significant differences in hemoglobin level between children with light-, moderate- and heavy-intensity hookworm infections (Table 3). For *A. lumbricoides* and *S. stercoralis*, no significant differences in hemoglobin levels between infected and uninfected children were observed. Children infected with hookworm had a significantly lower mean Z score for acute nutritional status compared to uninfected children (-0.25, 95% CI -0.45 - -0.06 vs. 0.12, 95% CI 0.00 - 0.25). This seemed to be due to the low mean Z score observed in children with light- and moderate-intensity hookworm infections as children with heavy-intensity hookworm infections did not show a lower mean Z score compared to uninfected children (Table 3). However, the low number of children with moderate and heavy-intensity hookworm infections in our study population (n=6 and n=9 respectively) precludes firm conclusions on the association between hookworm infection intensity and morbidity. Children with an *A. lumbricoides* infection showed a lower mean Z score for acute nutritional status compared to uninfected children, but this was only significant for moderate-intensity *A. lumbricoides* infection (mean Z score uninfected vs. moderate-intensity infection 0.07 (95% CI -0.06 - 0.20) vs. -0.54 (95% CI -1.00 - -0.07)).

**Table 3:** Association of infection intensity with indicators of acute nutritional status and hemoglobin values.

**Helminth/categories of intensity**	**n (%)**	**Weight-for-height or BMI-for age Z score**	**Hemoglobin (g/dL)**
		Mean (95% CI)	Mean (95% CI)
Hookworm			
Uninfected	279 (71.6)	0.12 (0.00 - 0.25)	14.0 (13.6 - 14.3)
Infected	111 (28.4)	-0.25 (-0.45 - -0.06)	12.8 (12.5 - 13.2)
Light	96 (24.6)	-0.34 (-0.55 - -0.14)	12.8 (12.4 - 13.2)
Moderate	6 (1.5)	-0.36 (-1.61 - 0.89)	12.7 (9.7 - 15.6)
Heavy	9 (2.3)	0.71 (0.09 - 1.33)	13.6 (12.3 - 14.9)
*Ascaris lumbricoides*			
Uninfected	286 (73.4)	0.07 (-0.06 - 0.20)	13.6 (13.2 - 13.9)
Infected	104 (26.6)	-0.12 (-0.32 - 0.09)	13.5 (13.1 - 13.9)
Light	52 (13.3)	-0.05 (-0.31 - 0.21)	12.7 (11.9 - 13.5)
Moderate	20 (5.1)	-0.54 (-1.00 - -0.07)	12.9 (11.7 - 14.1)
Heavy	32 (8.2)	0.04 (-0.37 - 0.46)	14.2 (13.8 - 14.7)
*Strongyloides stercoralis*			
Uninfected	359 (92.1)	0.04 (-0.07 - 0.15)	13.6 (13.3 - 13.9)
Infected	31 (7.9)	-0.24 (-0.60 - 0.13)	13.0 (12.2 - 13.8)
Light	11 (2.8)	-0.20 (-0.83 - 0.43)	13.0 (12.2 - 13.9)
Moderate	7 (1.8)	-0.25 (-1.38 - 0.88)	12.0 (9.5 - 14.4)
Heavy	13 (3.3)	-0.26 (-0.84 - 0.33)	13.6 (12.0 - 15.0)

Of the 390 included children, 142 (36%) were infected with multiple parasite species. Eighty-nine children (23%) were infected with two different parasite species, 46 children (12%) were infected with three species and seven children (2%) were infected with four different parasite species. In multivariate analysis including age, sex, indicators of acute and chronic malnutrition, region and previously administered anti-helminthic treatment as independent variables, the prevalence of polyparasitism was highest in the Amazon Region compared with the Orinoco Delta (OR 0.47, 95% CI 0.28 - 0.78) and the Carabobo State (OR 0.50, 95% CI 0.29 - 0.89). Multivariate logistic regression analysis did not show significant associations of polyparasitism with age, sex, nutritional status or the administration of anti-helminthic treatment in the past.

## Discussion

This large survey performed in school-age children from one non-indigenous and two indigenous populations in rural Venezuela showed high prevalence rates of parasitic infections in all three regions. The frequency of hookworm and *S. stercoralis* infections was highest in the Amazon Region while Warao and Carabobo State children showed more *A. lumbricoides* and *T. trichiura* infections. The prevalence of *G. lamblia* was around 20% in all three regions. The prevalence of malnutrition varied from 13% in the Carabobo State to 84% in the Amazon Region. In multivariate analysis including all three rural regions, acute nutritional status was significantly associated with helminth infections while chronic nutritional status was significantly associated with the presence of *G. lamblia*. Hemoglobin levels were significantly decreased in children with a hookworm infection and a significant positive association of hemoglobin levels with the height-for-age Z score, an indicator of chronic malnutrition, was observed in multivariate analysis. 

Studies performed in school-age children residing in other indigenous and non-indigenous rural areas of South America also showed high intestinal parasitic infection prevalence rates of 50% to 70% [36-39]. Differences in prevalence rates of intestinal parasitic infections between populations can be explained by behavioral, geographical and host genetic factors [40-42]. A behavioral risk factor found to be associated with intestinal parasitic infections is open field defecation, which is practiced in 40% of the rural Venezuelan population [43]. In particular, high *A. lumbricoides* and *T. trichiura* prevalence rates have been observed in communities where people defecate in open fields, bushes or bodies of water around their houses [44,45]. In the Carabobo State and Orinoco Delta, people defecate in the bushes or water surrounding their houses which could be an explanation for the observed higher *A. lumbricoides* and *T. trichiura* prevalence in these regions compared to the Amazon Region, where people defecate in specific areas further away from their *shabonos*. In urban areas of Venezuela, improved sanitation has led to a lower prevalence of helminth infections [18]. Besides improvement in household hygiene, health education adjusted to local ethnomedical knowledge and practices is also important for the reduction of parasitic infections, as demonstrated by a recently performed study in an Amerindian population from the Bolivian Amazon [37]. Wearing clothes and footwear can protect against skin penetration of helminths. In the Peruvian Amazon Region, *S. stercoralis* infection was significantly associated with not wearing shoes (OR 1.89, 95% CI 1.10-3.27) [46]. The high prevalence of skin-penetrating helminth infections we observed in the Amazon Region could be related to the fact that Yanomami children do not wear clothes or shoes, while Warao and Carabobo State children generally wear clothes and often wear shoes.

Environmental factors can also affect the dissemination and distribution of intestinal parasites in human communities. *A. lumbricoides* and *T. trichiura* are sensitive to high land surface temperatures and a lower prevalence of these helminths has been observed in regions where the maximum land surface temperature exceeds 40°C. In contrast, hookworm infection remains prevalent throughout the upper end of the thermal range [47,48]. The Amazon rainforest has a tropical climate with temperatures generally higher than those recorded in the Carabobo State and the Orinoco Delta, where winds decrease the temperature, especially in the afternoon and during the night. Similar to our observations in Yanomami children from the Venezuelan Amazon, in indigenous children from the Bolivian and Peruvian Amazon, hookworm infection rates were at least 3-fold higher than infection rates of *A. lumbricoides* and *T. trichiura* [37,49]. Hookworm seems to survive better in the continuously hot climate of the Amazon rainforest than *A. lumbricoides* and *T. trichiura*. This may be explained in part by the ability of mobile larvae to migrate to more suitable thermal conditions. Whereas hookworm larvae stages have some limited motility and can move downward into the soil, thereby avoiding desiccation, the ova of *A. lumbricoides* and *T. trichiura* are non-motile, and high surface temperatures will result in ova dying from desiccation [50]. The relationship between environmental conditions and survival of parasites may lead to seasonal variation in the prevalence of intestinal parasitic infections. In general, more children are infected with soil-transmitted helminths and *G. lamblia* in the rainy season while less acquisition of infections takes place in the dry season [51-53].Seasonal variation in parasitic infection prevalence could have influenced our study results as children in the Amazon Region and Carabobo State were included during the dry season while children in the Orinoco Delta were included over a period of 12 months. The inclusion of children in both the dry and the rainy season in the Orinoco Delta may have led to a relatively higher recorded rate of infection with soil-transmitted helminths and *G. lamblia* in children from this area. However, it is likely that an all-year-round transmission with little distinction between dry and rainy seasons occurs in this river delta as infective stages of intestinal parasites are present in the pools of river water that surround houses during the dry as well as the rainy season. An additional analysis including only children from the Orinoco Delta did not show significant differences between prevalence rates in children included during the rainy season compared to those included during the dry season for any of the soil-transmitted helminths or *G. lamblia* (median p-value 0.33, all p>0.05). Year-round transmission of hookworm was also observed in the riverine coastal communities in the delta of the Niger River in Nigeria [54].

As data demonstrating differences in parasitic infection prevalence between ethnically different populations are gathered, the need to better understand the role of human genetics and host immune response in susceptibility to parasitic infections becomes clear. Results of epidemiological studies suggest that up to 44% of the variance in worm burden is explicable by genetic effects, compared to 3% to 14% of variance explicable by environmental effects [55]. Ramsey et al. identified polymorphisms associated with *A. lumbricoides* infection in Venezuelan children from Coche Island [56]. In Yanomami and Warao children, a genetic susceptibility to tuberculosis associated with an altered immune response has been suggested [57,58]. Whether genetic factors play a role in the region-specific pattern of intestinal parasitic infections in Venezuela remains to be investigated.

Besides behavioral, geographical and genetic factors, variation in the detection methods used could have played a role in observed differences in parasitic infection prevalence rates between regions. Chhakda et al. used three different fixatives, namely Kato-Katz, Baermann and SAF techniques, in the same set of single fecal samples taken from Cambodian school-age children [59]. The percentage of children infected with *S. stercoralis* detected by Baermann and SAF fixation in their study was 20% and 3%, respectively. According to the World Gastroenterology Organization (WGO), the Baermann technique is regarded as the gold standard for the detection of *S. stercoralis* in stool [60]. Therefore, the difference in *S. stercoralis* prevalence between the three regions in our study could be due to a difference in technical methods, as Baermann was only used in the Amazon Region. However, in an earlier study performed in the Orinoco Delta, the prevalence of *S. stercoralis* infection assessed by the Baermann technique was very low [61]. In El 25, the Baermann technique has not been used in previous surveys. Hookworm prevalence in the Amazon Region was more than ten times higher than in the other two regions. As only a 1.3-fold difference was observed between Kato-Katz and SAF analyzed samples in the Cambodian study [59], it is very unlikely that the observed difference in our study was solely due to methodological issues. Another methodological difference to take into account in the interpretation of the results of our study was the difference in the number of samples taken from each child. In the Yanomami children only one fecal sample was collected while two consecutive samples were collected from the Warao and Carabobo State children. The examination of two instead of only one Kato-Katz smear increased the observed prevalence of hookworm with 160% in schoolchildren in Tanzania while the percentage of detected *T. trichiura* and *A. lumbricoides* showed a less than 1.5-fold increase when two samples were taken [62]. Since we observed the highest prevalence of hookworm in the Amazon Region where only one fecal sample was collected from each child, it is likely that the observed difference in hookworm prevalence between regions reflects a true difference. Also, the difference in *A. lumbricoides* prevalence between children from the Amazon Region and the Carabobo State was more than 2-fold and therefore probably not solely due to the collection of two samples in the Carabobo State. For *G. lamblia* infections, no differences in prevalence rates when one, two or three samples were collected were seen in a study including children from Colombia [63]. 

We observed significantly higher rates of eosinophilia in children infected with helminths, in particular *S. stercoralis* and hookworm, compared to uninfected children. Eosinophils are elicited by Th2 effector cells and involved in the initiation and regulation of Th2 immunity [64]. Intestinal helminths are reported to induce a Th2 type immunity in the host and evidence suggests that the Th2 immune response may play a crucial role in reducing the severity of acute disease upon helminth infection [4]. Up-regulation of Th2 responses including eosinophilia by helminth infection can suppress the production of a Th1 immune response which is important to combat intracellular pathogens such as *Mycobacterium tuberculosis* and the Human Immunodeficiency Virus (HIV) [65,66]. The association between helminth infections and eosinophilia in rural Venezuelan children indicates an effect of helminths on host immunity. As prevalence rates of tuberculosis and HIV are rising in children residing in rural areas of Venezuela [67-69], it is important to consider the immunomodulatory role of helminth infections in these diseases.

### Association of parasitic infections and anemia with nutritional status

We observed a significant association of *G. lamblia* and helminth infection with stunting and wasting status respectively. *G. lamblia* infection was also associated with stunting, but not with wasting, in children from other South American countries [70-72]. In a recently performed study including Ethiopian school-age children, helminth infections were also significantly related to diminished BMI-for-age Z scores while height-for-age Z scores did not differ significantly between children with and without helminth infections [12]. In schoolchildren from Nigeria and Uganda helminth infections were associated with both wasting and stunting [13,14]. Stunted children generally show increased intestinal permeability levels resulting in a poor gut function and malabsorption [73]. *G. lamblia* infections are more commonly associated with intestinal permeability than helminth infections, which is a possible mechanism for the association of *G. lamblia* infections with chronic malnutrition [73,74]. In contrast to studies from South America, a significant association of *G. lamblia* infection with wasting and of trichuriasis with stunting was observed in Asian children [75-77]. The etiology of malnutrition is complex and a full understanding of its causes and the role of different parasite species requires more detailed investigations into immune factors and gene-environment interactions. 

Due to the cross-sectional study design, it was unknown whether a poor nutritional status is a risk factor for infection with intestinal parasites or whether intestinal parasitic infections affect growth leading to growth deficits and a poor nutritional status. Longitudinal studies have demonstrated that the relationship between intestinal parasitic infection and malnutrition is probably bidirectional. The nutritional status of school-age children from a slum area of the capital city of Venezuela, Caracas, improved significantly after regular anti-helminthic treatment for a year, suggesting that helminth infections affect growth. On the other hand, the finding that the degree of re-infection by helminths after the end of administration of anti-helminthic treatment in this study was significantly higher in malnourished children suggests that a poor nutritional status enhances the acquistion of helminths [78]. In rural Guatemalan children receiving anti-helminthic and anti-protozoan treatment no effect of anti-helminthic administration on growth was observed, but increased growth was significantly related to eradication of *G. lamblia* [79]. A Cochrane meta-analysis showed an increase in weight gain after a dose of deworming drug given to children infected with worms (mean difference of 0.58 kg, 95% CI 0.40 - 0.76). However, it is not known whether deworming of groups of children without prior diagnosis also leads to an increase in weight [80]. Concordant treatment of *G. lamblia* infections seems to be important, as shown by Northrop-Clewes et al. In their Bangladeshi study they reported a significant increase in the prevalence of *G. lamblia* in a group of children treated with anti-helmintics, which was significantly associated with higher intestinal permeability (p<0.001) and a short-term reduction in weight (p=0.02) in infected individuals [81]. In order to have a long-term effect on nutritional status and growth, anti-parasitic drugs need to be integrated with nutritional interventions such as micronutrient supplementation [82]. Anti-helminthic treatment may also improve hemoglobin values, but again there is insufficient evidence on this matter according to the recently performed Cochrane review [80]. We observed a significant positive association of hemoglobin levels with height-for-age Z scores. Low hemoglobin concentrations in stunted children were also observed in school-age children in Cote d'Ivoire and China [7,83]. Hemoglobin levels were significantly lower in children infected with hookworm in our study population as well as in other populations [3,84]. Anemia in childhood can have serious long-term health effects including reduced cognitive and social-emotional development [85]. Most children in our survey suffered from light-intensity hookworm infections (Table 3). This is probably the reason for the absence of a significant difference in hemoglobin level between light-, moderate- and heavy-intensity hookworm infections. A systematic review summarizing intensity-stratified associations between hookworm infection and anemia showed that moderate- and heavy- but not light-intensity hookworm infections were associated with lower hemoglobin levels in school-age children [9]. 

The natural history of morbidity in helminth infections is such that the acute inflammation produced by the initial helminth infection is largely reversible. However, as the number of worms increases, the inflammation progresses into a chronic stage with irreversible sequelae [10]. The low number of children with moderate- or heavy-intensity infections in our survey may indicate that inflammation has not yet progressed into a chronic stage in the majority of children. Large-scale distribution of anti-helminthic drugs could prevent irreversible and permanent sequelae in these children [10]. The WHO recommends the implementation of deworming programmes centered on school delivery because of the large burden of morbidity and concomitant developmental consequences of helminth infections for school-age children [86] as well as the relative ease of access to children in poor rural areas through schools and the cost-effectiveness of school-based deworming [87,88]. Although advocacy for a school-based approach to the control of helminth infections and the morbidity they induce has been made by many over the past decade [89-91], data from anthropometric surveys in more than 50 countries worldwide suggest that, in order to reduce undernutrition, deworming programs should be implemented before 24 months of age [92]. Young preschool children are at a critical stage of growth and development which increases their vulnerability to the detrimental effects of intestinal parasitic infections. Additional studies investigating the association between intestinal parasitic infections and nutritional status in preschool children in Venezuela are needed.

The present study shows that helminthiasis and giardiasis are associated with a high prevalence of acute and chronic malnutrition respectively in rural communities in Venezuela. This is the first study comparing parasitic infection prevalence and nutritional status in three geographically dispersed areas resided by ethnically different populations in a South American country. Behavioral, environmental and genetic factors may play an important role in the observed differences in prevalence rates of parasitic infections between the regions. These factors should be taken into account when prevention and control programs are developed.
